# The benefits of meditation and mindfulness practices during times of crisis such as COVID-19

**DOI:** 10.1017/ipm.2020.38

**Published:** 2020-05-14

**Authors:** C. Behan

**Affiliations:** Department of Psychiatry, Royal College of Surgeons, Dublin, Ireland

## Abstract

Meditation and mindfulness are practices that can support healthcare professionals, patients, carers and the general public during times of crisis such as the current global pandemic caused by COVID-19. While there are many forms of meditation and mindfulness, of particular interest to healthcare professionals are those with an evidence base such as mindfulness-based stress reduction (MBSR). Systematic reviews of such practices have shown improvements in measures of anxiety, depression and pain scores. Structural and functional brain changes have been demonstrated in the brains of people with a long-term traditional meditation practice, and in people who have completed a MBSR programme. Mindfulness and meditation practices translate well to different populations across the lifespan and range of ability. Introducing a mindfulness and meditation practice during this pandemic has the potential to complement treatment and is a low-cost beneficial method of providing support with anxiety for all.

## Introduction

During the global pandemic, we have all had to change how we live and work. Healthcare workers may be overwhelmed, busy, fearful, coping and dividing their lives. Patients with pre-existing anxiety, depression or psychosis may feel overwhelmed by additional worry or fear. People with substance abuse may turn increasingly to whatever they can obtain to manage their own addiction. Carers may have an additional burden of care with loss of any time to themselves and loss of supports. Household units are living in pressured environments, not used to spending so much time in small spaces. Children and adolescents have lost the structure provided by school and may have their own worries and fears, with loss of support external to their own families. Older people have been cocooned away and may have lost not only external support but may have the perception that once you are over 70 you are deemed vulnerable and no longer useful. Add financial and employment instability into the mix and we have a society structure that is challenged. Yet, despite the despair, the fear and the anxiety, there have been glimpses of a world with an increased sense of community and kindness.

### Meditation and mindfulness

Meditation and mindfulness are terms that have crept into mainstream culture. These terms are often used interchangeably, but there are subtle differences between the two. Meditation usually refers to a formal practice that can calm the mind and enhance awareness of ourselves, our minds and our environment. Meditation in its many guises has been practised over millennia by diverse groups of people in many different traditions. Previously practised primarily in the Eastern traditions, meditation has spread into Western society and is increasingly being used as a therapeutic modality. ‘Mindfulness’ as a term has become ubiquitous in recent times. Mindfulness simply means being aware of the present moment. Meditation comes under the umbrella of ‘mindfulness’ which is a broader concept. Formal meditation practices include mindfulness of breathing, compassion or loving kindness-focused meditation, the use of mantras or phrases as the focus for meditation, amongst many others.

Underlying each of the different meditation techniques is a simple coming to awareness of the present moment. Being aware of what is happening in the present moment allows the individual to observe what is arising and what is falling away. By doing this and by allowing thoughts to come and go without attachment, without trying to hold on to them, we learn that calm and stillness follows. We come to know our own minds over time and to be aware of patterns of thinking that habitually arise. The key is to gently catch a spiral of thoughts, mind flurry or mind chatter, and observe, noting ‘worry’, ‘lists’, ‘craving’, ‘fear’, and allow the spiral to gently fall away without judgement. Useful techniques in different forms of meditation include mindfulness of breathing (using the breath as an anchor to the present moment), compassion-focused meditation (using loving kindness, and awareness of others’ and our own suffering to be in the present moment), the body scan (being aware of each part of the body in turn as an anchor for the present moment and for where we hold tension and stress in our bodies). Other forms include the use of mantras or phrases to focus attention to the present moment, or walking meditation where the entire focus is on awareness of our feet in contact with the earth and grounding to the present moment. Over time, regular practice of mediation allows individuals to react to their environment and anything that arises in the course of their day with more calm and equanimity. Studies of people who have meditated over the long-term show changes in areas of the brain concerned with stress and anxiety (Afonso *et al*. 2020). The prefrontal cortex, the cingulate cortex and the hippocampus show increased activity, and the amygdala shows decreased activity consistent with improved emotional regulation. Other studies have shown that evidence-based therapies such as MBSR also show similar brain changes to those with traditional meditation practice (Gotink *et al*. 2016).

As the term ‘meditation’ is so broad, and its therapeutic uses encompass many conditions including pain, mental health and somatic conditions, research into such heterogenous modalities and outcomes has been difficult to conduct in a systematic scientific manner. Recently, there have been more studies of better quality resulting in randomised controlled trials and systematic reviews. Practices such as MBSR and mindfulness-based cognitive therapy (MBCT) have developed from formal meditation practices. These are modularised practices that are feasible to teach and lend themselves more easily to evidence-based practice research. Both are 8-week-long programmes of 2 hours duration a week, with intensive 1-day teaching midway through the programme, and with home practice. MBSR, originally developed by John Kabat-Zinn in the 1970s, adapts formal meditation practices to give a more generalised approach to mindfulness, and MBCT was subsequently developed with more of a focus on depression using a blend of cognitive and mindfulness approaches.

The predominant symptoms presenting at this time in many of our patients, in society and in ourselves are anxiety, overwhelm and despair. These are natural sequelae of existing in the time of a global pandemic of what will be an uncertain duration. Systematic reviews of meditation-based tools such as meditation using focused attention, MBSR and MBCT have shown reduced anxiety (Montero-Marin *et al*. 2019), depression and post-traumatic stress disorder (Khusid & Vythilingam, 2016), stress (Juul *et al*. 2020), blood pressure, cortisol levels and other physiologic markers of stress (Pascoe *et al*. 2017). Having a regular meditation practice can benefit people working in the health service (Lomas *et al*. 2018) in addition to the benefits for patients, the general population and those with pre-existing mental illness. Meditation techniques are easy to learn, easy to support online (Krusche *et al*. 2013; Chadi *et al*. 2018, 2020; Champion *et al*. 2018), can be done as an individual and there are benefits from a group practice. Meditation techniques can be adapted for adults, children, teens and those with intellectual disabilities (Chadi *et al*. 2018; Singh & Hwang, 2020). While MBSR and MBCT are usually taught face to face by a certified professional, there has been increased investigation of the use of meditation apps and online eHealth and Telehealth in delivering such interventions (Champion *et al*. 2018; Huberty *et al*. 2019). There are a large number of meditation apps available, such as Calm, Headspace and Insight Timer, that people can use to support their own meditation practice. In such times as the COVID-19 pandemic, these and other techniques may be useful to support people. Preliminary studies show benefit to people in areas such as sleep from having a mindfulness practice (Zheng *et al*. 2020). There are also a number of organisations offering opportunities to create a pause in our lives such as the Royal College of Physicians (#pauseforapoem), reportedly with some interest and uptake. Places with established traditions of teaching others secular, non-secular and non-healthcare-based meditation and mindfulness such as The Sanctuary and the Dublin Buddhist Centre are offering online learning meditation sessions at reduced cost or free. Anecdotally from these centres, there has been a large increase in uptake of these classes. As this pandemic is early in its infancy, there is little peer-reviewed research to evaluate the effects of these offerings, but they are certainly helpful based on previous studies and could provide fruitful areas of research interest.

Crises such as the COVID-19 pandemic have shown that change is the only constant. Meditation and mindfulness can offer a helpful way to live with this constant change. MBSR programmes already extant within services can be adapted for online delivery. Meditation apps and online classes can be recommended to patients. Both learning and having a regular meditation practice ourselves can only benefit our patients and ourselves. Meditation and mindfulness are useful skills that can help us to sit with our fears and our circumstances and to observe that like our thoughts, this period in our lives too shall pass.
